# The Use of Microfluidics in Cytotoxicity and Nanotoxicity Experiments

**DOI:** 10.3390/mi8040124

**Published:** 2017-04-12

**Authors:** Scott C. McCormick, Frederik H. Kriel, Angela Ivask, Ziqiu Tong, Enzo Lombi, Nicolas H. Voelcker, Craig Priest

**Affiliations:** 1 Future Industries Institute, University of South Australia, Mawson Lakes Blvd., Mawson Lakes, 5098 SA, Australia; scott.mccormick@mymail.unisa.edu.au (S.C.M.); Erik.Kriel@unisa.edu.au (F.H.K.); angela.ivask@kbfi.ee (A.I.); tommy.tong@monash.edu (Z.T.); Enzo.Lombi@unisa.edu.au (E.L.); nicolas.voelcker@monash.edu (N.H.V.); 2 Monash Institute of Pharmaceutical Sciences, Monash University, Parkville, 3052 VIC, Australia

**Keywords:** microfluidics, cytotoxicity, nanotoxicity, nanoparticles, screening, 87.17.UV Biotechnology of cell processes, 87.85.dh Cells on a chip

## Abstract

Many unique chemical compounds and nanomaterials are being developed, and each one requires a considerable range of in vitro and/or in vivo toxicity screening in order to evaluate their safety. The current methodology of in vitro toxicological screening on cells is based on well-plate assays that require time-consuming manual handling or expensive automation to gather enough meaningful toxicology data. Cost reduction; access to faster, more comprehensive toxicity data; and a robust platform capable of quantitative testing, will be essential in evaluating the safety of new chemicals and nanomaterials, and, at the same time, in securing the confidence of regulators and end-users. Microfluidic chips offer an alternative platform for toxicity screening that has the potential to transform both the rates and efficiency of nanomaterial testing, as reviewed here. The inherent advantages of microfluidic technologies offer high-throughput screening with small volumes of analytes, parallel analyses, and low-cost fabrication.

## 1. Introduction

Toxicity studies are important in biochemical and medical research, and essential prior to the commercial use of newly developed chemicals and nanomaterials. The health and safety of researchers, production workers, end-users, and bystanders who may come into contact with new or innovative products, and any secondary products that may arise from the degradation of such products, is of great concern to government regulatory bodies and society as a whole [1,2,3]. The benefits of the effective toxicity screening of chemicals and nanomaterials prior to their commercialization include better community health outlooks, reduced costs (healthcare and/or compensation payments), and faster paths to the market for new non-toxic products. Currently, biochemical and medical products are subjected to extensive testing before adoption or commercialization, but this is costly in terms of labour, time, and money. Many companies are specifically set up to assist with performing the biocompatibility (i.e., toxicology) screening for new formulations. Analysts predict that the in vitro toxicity testing market value will reach approximately 10 billion dollars in 2017 [4]. Thus, new technologies for toxicity screening are attractive for their perceived economic, social, and environmental benefits.

Cytotoxicity analysis at a cellular level is concerned with how a given toxic chemical affects a given cell’s physical structure (e.g., membrane integrity) and its ability to viably replicate without damage to the daughter cell’s genetic code or normal functionality [5]. Thorough cytotoxicity screening of a chemical requires the studied toxicant to be tested against different cell types, and is generally performed in static fluid in well plates. This approach requires laborious liquid handling, long hours of incubation, and large reagent volumes. The idea of an all-encompassing cytotoxicity test is a daunting task, as the adult human body contains trillions of eukaryotic cells with different phenotypes and functionality [6] and, according to Vickaryous [7], the number of unique cell types is 411, including 145 types of neuronal cells. The many different cell types in the human body makes the effective in vitro screening of potential toxic effects an enormous challenge, further complicated by the types of analysis required (e.g., viability, cell metabolism, and biochemistry). The growing number of novel chemicals and materials that are being suggested for various commercial applications multiplies the size of the challenge and calls for much faster and cheaper methods.

Nanotoxicity is an important subgroup of toxicity which considers the damaging effect of nanomaterials on cells. The first reports on the toxicity of nanomaterials on mammalian biology were reported in the 1990’s by Jani et al. [8] and Penney et al. [9]. Now, an awareness of the potential toxicity of those materials has reached beyond the scientific community to include regulators and consumers, who, in many cases, are not equipped with enough information to guide their decisions. All the while, more nanomaterials are being created and incorporated into consumer products, from sunscreens [10,11] and cosmetics [2,12], to antibacterial and antifouling coatings [13,14,15]. A major apprehension pertaining to the exposure of the human body to nanoparticles is their physical size (typically 1–100 nm), which can allow them to enter cells via pathways that naturally transport biological and chemical species [5]. Many nanoparticles can form free radicals and reactive oxygen species (ROS) [16] from surrounding molecules, due to their increased reactivity and high surface area, which have the ability to cause damage to cellular membranes and proteins within cells, leading to inflammation and oxidative stress [17,18]. Nanomaterials can also disassociate into ionic species upon reacting with biological tissue and fluids such as gastric juices, which can lead to the release of reactive ions that can damage the cellular environment and cause toxic effects [19,20]. Genotoxicity can occur if a nanoparticle interferes with the delicate process of DNA transcription and replication, potentially knocking out one or more genes from the sequence and causing a range of negative effects, such as apoptosis (in which case the cells die off) [21,22,23] or mutation, which can lead to the cells becoming cancerous [24].

Meaningful nanotoxicity studies require high-throughput screening methods, as the toxic effects of a nanomaterial can be dependent on the core composition, size, shape, and surface modification that material possesses, suggesting that a high number of materials should be tested. The many different cell and tissue types of the human body can react in different ways to any given nanomaterial, meaning that an ideal toxicology screen should test every unique cell type. Thus, the desire to create a fast, stable screening process for the maximum number of combinations of nanoparticles and human cells possible is of great interest to the industry and health sectors. Where practical, these methods will enable the determination of safety exposure levels and maintain the health of both workers in the nanomaterial industries and the end-users of products containing nanomaterials. To this end, microfluidic approaches to toxicity screening have been investigated and are the topic of this review.

## 2. Nanomaterial Exposure Pathways in Biology

**Figure 1:**
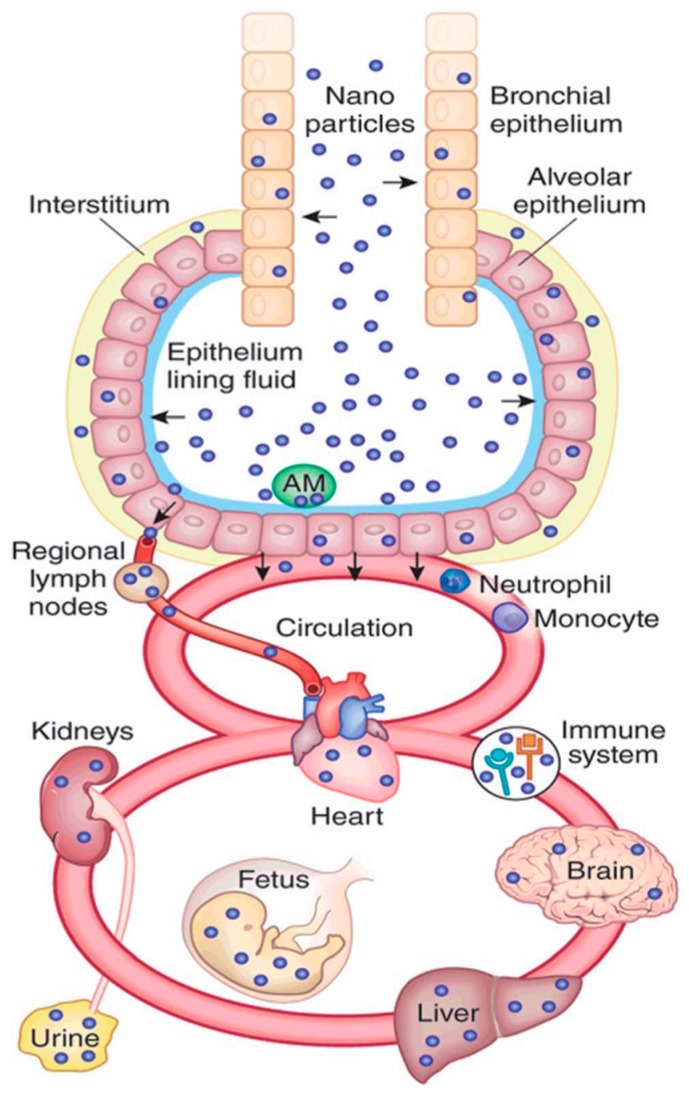
Schematic diagram of nanoparticle pathways through the human body after inhalation, Reprinted by permission from Macmillan Publishers Ltd: NATURE BIOTECHNOLOGY Kreyling, Hirn [28], copyright 2010.

Nanomaterials can be taken up into a living body via the natural internalization pathways of ingestion, inhalation, or dermal uptake [25], or through direct injection if used in nanomedicine [26], and can pose a substantial risk to the viability of a cell, depending on the nature of their interaction with living cells. Once inside the body, nanomaterials can transmit from one tissue to another via the bloodstream and the surrounding tissues, potentially migrating into other organs such as the kidney or spleen. Once they enter the body, they come into contact with the body’s cellular structure and potentially gain entrance into the cells themselves [27]. A diagram of nanoparticles and the bodily areas that they are able to access after uptake via inhalation is shown in Figure 1. The uptake of nanoparticles to the body and different organs is particle size dependent. As an example of size-dependency on the uptake of nanoparticles, it was shown by Jani et al. [8] that when a range of polystyrene particles ranging from 50 nm to 3 µm was introduced to a rat model via ingestion pathways, no particles above 100 nm reached the bone marrow and none larger than 300 nm were present in the bloodstream, whereas the 50 nm and 100 nm particles were absorbed at rates of 34% and 26%, respectively, into the liver, spleen, blood, and bone marrow tissues.

Nanoparticles can pass through the cellular membrane via the passive transport mechanics of diffusion and osmosis, requiring no activation energy [29,30]. Alternatively, nanoparticles can be taken into the cell via active transport mechanisms, in which carrier proteins or ionic pumps within the cell membrane attach to the particle and use energy to move them across the cell membrane into the cytoplasm [27]. Inorganic nanoparticles that require this mechanism to cross the membrane are often blocked from entering cells, unless they are coated with a biomolecule (such as transferrin) that facilitates their uptake by the carrier proteins, as was shown in Yang et al. [31] in the case of transferrin-conjugated gold nanoparticles.

Nanoparticles that do not enter the cell via membrane diffusion or through membrane pores can still be transported into the cell via endocytosis [32]—that is, the cytoplasm of the cell extends around a particle and engulfs it, forming an endocytic vesicle that retains them in the inner cytoplasm of the cell. From here, the particles can either: escape the vesicle and remain in the cytoplasm; persist in the vesicle and be consumed by a lysosome (an organelle full of enzymes that serve to digest foreign bodies that exist in the cytoplasm); or combine with other vesicles to form a multi-vesicle endosome contained in a secondary membrane, which stabilizes and contains the individual vesicles [30].

Certain nanoparticles possess the ability to alter or bypass the membrane permeability, depending on their ionic potential or their shape. Nanoparticles that are shaped with sharp points or edges can mechanically damage the cell membrane, creating temporary nanochannels through which they can enter the cytoplasm [33]. This can be exploited to create drug delivery mechanisms by coating nanoparticles such as carbon nanotubes with biocompatible molecules which attach and enter cells, or manufacturing nanoneedles from materials such as silicon or polymers that can mechanically puncture cells to deliver drugs directly into the cytoplasm [34,35]; however, toxic nanomaterials could very easily enter cells by the same mechanism and induce cytotoxic effects.

The above pathways and cellular interactions are complex and very sensitive to the size, shape, chemistry, and surface charge of the nanomaterials, meaning that the importance of a high-throughput evaluation of nanotoxicity is growing commensurate with the rapid development of nanotechnology. In recent years, microfluidics technology has resulted in large impacts on the cytotoxicity screening of nanoparticles. This will be discussed in the following section, to underpin the later discussion of nanotoxicity screening using microfluidic chips.

## 3. Microfluidics in Cytotoxicity Screening

Microfluidics has garnered a great deal of interest in the field of in vitro cytotoxicity screening. Combining biological engineering with microfluidics is termed cell-laden microfluidics, in which living cells are affixed within a microfluidic channel, exposed to various chemical species in a flowing medium, and assayed to determine their post-exposure viability using viability assay dyes. Viability assay dyes are chemical compounds that are taken up into either living or dead cells, or bind to cell-death markers that are released outside of dead cells. They contain a fluorescent or coloured moiety that can be detected and quantified using optical detection methods [25,36]. These fluorescent methods only require a very small amount of dye per cell, and are therefore reasonable candidates for miniaturization into a microfluidic platform.

The use of microfluidic platforms in cytotoxicity screening is desirable due to a number of factors, i.e., small sample volumes [37], reduced costs [38,39], a controlled and reproducible laminar flow, and the ability to functionalize (e.g., antibodies) (36) or structure (e.g., compartments) [37] microchannels to produce a varied microarray of multiple cells. It also seeks to address the current paradigm of static well-plate testing that, as Cunha-Matos et al. stated, “these procedures provide averaged results, do not guarantee precise control over the delivery of nanoparticles to cells and cannot easily generate information about the dynamics of nanoparticle-cell interactions and/or nanoparticle-mediated compound delivery” [40]. There are multiple variations on cell-laden microfluidic methods and protocols, some of which are detailed in the following sections.

### 3.1. Cell Capture and Immobilisation

**Figure 2:**
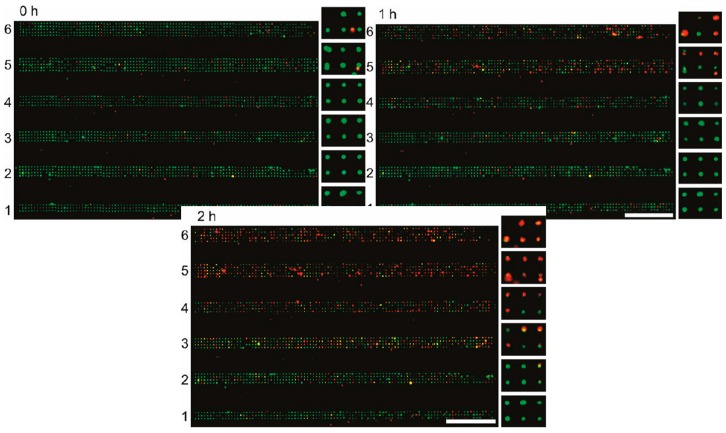
Six parallel channels containing single-cellular microwell arrays after exposure to gradients of KCN, green indicates viable cells and red indicates lysed cells, scale bar = 1 mm. Adapted with permission from Analytical Chemistry 83(10) Hosokawa, Hayashi [37]. Copyright 2011 American Chemical Society.

The concept of cell-laden microfluidics requires cells to be immobilized inside the channel in such a way that they can be analysed under precisely controlled (on-chip) conditions. A device that marries the concept of well plates and microfluidic flow for single-cell capture was designed by Hosokawa et al. [37]. The microfluidic device was assembled on top of a laser-perforated polymer microcavity array, which was created with conical cavities measuring 2 µm at the surface. This was assembled on top of a vacuum line which applied negative pressure to pull a cell suspension through the channel and produced a microarray of single cells as they settled in the perforations. Further, an on-chip chemical gradient generator was used to treat six channels with unique concentrations of a potential toxicant (potassium cyanide), which were subsequently stained with cell viability assay dyes (EthD-1, which stained exclusively the DNA of lysed cells), allowing the high-throughput screening of these different concentrations. The results of their experiments can be seen in Figure 2, where they showed increases in red fluorescence corresponding to greater numbers of lysed cells as the concentration of the toxicant increases.

A device that utilized a larger-scale microwell environment was produced by the group of Weibull et al. [41]. In order to investigate the behaviour of cells to a concentration gradient without interference from intercellular paracrine signalling from neighbouring cells, they developed a system in which single cells could be analysed. They fabricated a microscope-slide-sized microwell plate with 672 (14 × 48) 500 nL wells, in which only single cells would fit by attaching a grid of etched silicon to a glass slide, allowing for high-resolution imaging. They then layered three PDMS channel designs over the top of this microwell plate, which produced a concentration gradient generator in which a reagent from the top layer mixes with a diluent from the middle layer, and flows down to the reaction chamber in the bottom layer, before exposing the mixture to the cells beneath. Their device was able to culture bovine aortic endothelial cells in the silicon microwells and expose the cells to differing concentrations of saponin to induce cell death. Live/dead staining could be performed by exposing dyes through the same ports.

Cell traps are useful for their ability to analyse non-adherent single cells, but often adherent cells are used in experiments that are usually found in large contiguous layers. To achieve larger areas of cell attachment, cellular microarrays have been produced by printing antibodies onto a substrate, before assembling the microfluidic device and exposing the printed area to a culture of cells. These methodologies are suitable for the robotic “spotting” of antibodies or proteins, and the subsequent binding of the target cells to these scaffolds [42]. By printing antibody spots that are selective for unique cell types, the cells bind to the surface and form a microarray, allowing non-selected cells to continue flowing and either bind to another spot, or flow out as waste. A two-dimensional array that binds a flat patch of cells can be produced on a substrate with a modified inkjet printer [43] or micro-contact printer [44].

Our group has investigated the potential of microfluidics for a high-throughput screening methodology. In a recent paper by Tong et al. [42], a device incorporating five parallel laminar streams crossed with five perpendicular streams was investigated, in order to bind different cell types in a single device, and subsequently delivered different chemical treatments to the cells to create an orthogonal microarray. Antibody-antigen binding or extracellular matrix (ECM) protein binding was utilized in a 5 × 5 array with a microcontact printer to anchor cells to a glass substrate under microfluidic flow. A model cytotoxicity assay was performed using various levels of osmotic stress in different laminar streams, and fluorescein diacetate/propidium iodide viability assay dyes were applied to achieve a fluorometric readout of cell viability.

In a reversal of the concept of micropatterning to create a binding surface for cells flowing in media, the group of Leclerc et al. [45] used a PDMS microfluidic channel with the microstamps on the top channel wall to press down and crush any bound cells within the area of the stamp. By first culturing a layer of cells on the bottom channel surface, when the crushed cells were washed away by perfusion, they allowed new cells to grow in the affected area. This technique could allow for the long-term culturing of the same cell sample, thus enabling many concurrent tests to be carried out on the same chip.

In microfluidic systems where larger cell binding areas are required for analysis, it is possible to flow a bio-functional binding agent through a microfluidic device and coat the entire channel surface. Pasirayi et al. [39] utilized a multilayer device that sandwiched a 10 μm PDMS membrane between two PDMS channels, held together by two rigid polymethylmethacrylate (PMMA) cover plates. One of the PDMS channel sides contained a concentration gradient generator, while the opposite side contained cell culture chambers separated by valve arrays to prevent cross-contamination. The valves could be opened and closed by the application of a mild vacuum, allowing for fine control over the exposure and conditions in the cell culture chambers. They coated the chambers with an extracellular matrix (ECM) protein, fibronectin, and attached model breast cancer and liver cells. The cells were kept viable by perfusing fresh media. Finally, the cell culture chambers were exposed to an antibiotic, pyocyanine, via the concentration gradient generator to achieve a range of 0–100 μM, and a fluorescent live-cell assay was performed with Calcein AM. A combination of drugs, paclitaxel and aspirin, was also tested to identify potential synergistic toxic effects, and assayed in the same manner as the pyocyanine.

In recent years, advances in lithographic techniques and cellular gels have enabled the production of cell arrays in microfluidic channels post-assembly. The production of natural hydrogels based on in vivo ECM proteins, i.e., collagen, or very similar synthetic products like Matrigel^®^ (Corning, NY, USA) and alginate, has allowed for 3D scaffolds and structures that very closely mimic an in vivo cellular microenvironment [46]. Groups such as Toh et al. [47] have produced multiplexed 3D microfluidic cell culture systems in which primary hepatocytes could be cultured. Their chips utilized separating micropillars to divide the cell culture channels into a central cell culture area surrounded by two perfusion channels, and a linear concentration gradient generator delivered culture medium and drug solutions to the cells via the perfusion channels. They prepared a hydrogel of methylated collagen and terpolymer combined with hepatocytes that could be flowed through the microfluidic channels to settle in the cell culture area, which maintained function and produced albumin proteins. They tested five model hepatotoxic drugs using the concentration gradient generator and produced toxicity data by fluorescently staining the cells post-treatment.

Microfluidic chips can be used to study the ecotoxicity and cytotoxicity of prokaryotic cells. Yoo et al. [48] utilized a water-soluble photosensitive polymer to create patterns of bioluminescent bacteria inside a microfluidic chip. By flowing a mixture of the monomers of the photosensitive polymer and strains of genetically modified oxidative stress-induced bioluminescent *E. coli* through the channel and selectively exposing it to UV to cause gelling, they were able to bind the bacterial cells on the exposed areas and measure their luminescence intensities. A UV exposure of 10 mins was found to be short enough for bacterial cells to recover from the binding process, after which they could be used as a toxicity testing platform. Upon exposure to both hydrogen peroxide and phenol via the microfluidic flow, the bacterial cells underwent oxidative stress and presented a more intense luminescence in a dose-dependent manner, meaning that they could be utilized for chemical screening.

The emerging technology of organ-on-a-chip seeks to enable testing on human biomimetic environments, by producing three-dimensional cultures of human cells that possess a similar structure and shape as those in in vivo conditions. The group of Wagner et al. [49] utilized this beneficial technology by producing a microfluidic environment with multiple culture locations, connected by the flow pathway; in which they cultured both biopsied skin tissue and pre-grown liver microtissue aggregates. The chips were infused with just 300 μL of cell culture medium and sealed, with perfusion being provided by an on-chip micropump. The medium required only a 40% replacement at 12 h intervals for the first week of culture. To prove the usefulness of the devices in toxicity testing, they exposed the system to troglitazone, a drug with a known hepatotoxicity. A dose-dependent toxic response was detected by assaying the culture medium for glucose consumption and lactate production. There was a visible increase in the cytochrome concentration in the drug-exposed samples when the cells were immunostained after the device was disassembled. They also showed the potential of using the skin layer as an air-liquid interface for more realistic methods of applying topical drugs in future devices. Overall, this device shows the promise of multi-organ microfluidic devices for investigating specific uptake profiles and the run-on effects between different bodily organs.

Very few standardized microfluidic platforms are available for toxicology testing. One company called SynVivo [50] provides a standardized toxicity assay chip in which a ring of endothelial cells can be cultured around a choice of other tissue cells, i.e., cardiomyocytes and hepatocytes. Their platform enables optical and fluorescent imaging, as well as chemical assays such as an ROS assay, and has been shown to culture liver cells such that they successfully produce urea and responded to the toxicity of acetaminophen and doxorubicin. Platforms like these must become much more commonly produced if microfluidic cytotoxicity assays are to be accepted as standardized testing.

### 3.2. Channel Arrays and Laminar Flow

Microfluidics presents the ability to separate fluid streams from each other using physical barriers or the properties of laminar flow. One of the earlier examples of microfluidics’ use in cytotoxicity experiments came from the group of Ma et al. in 2008 [51], who fabricated channels in a quartz chip and two additional channel-containing PDMS layers attached to opposite sides. The chip was designed to test both the cytotoxicity and cellular metabolism of drugs in human liver microsomes (HLMs), which catalyze drug metabolism. HLMs were applied to the devices’ microwells in a homogenous sol-gel suspension, held in place by reversibly bonded PDMS. Liver carcinoma cells (hepG2) were cultured in chambers that were exposed to the metabolic products from the HLMs. The mixtures of liver-active drugs, acetaminophen, and phenytoin, in addition to the viability assay dyes, were introduced across the sol-gel columns via microfluidic flow. Viability was determined via fluorescence imaging, while drug metabolism was determined by UV absorbance spectroscopy, performed on the flowing media before it exited the device.

In order to show the reproducibility of microfluidic cytotoxicity experiments, multiple repeat experiments of a 64-chamber microfluidic chip were performed by Cooksey et al. [52]. Their cell culture chambers were arranged in an 8 × 8 pattern, and multiple different cell densities were seeded on fibronectin for an analysis of the expression of transfected destabilized green fluorescent protein (GFP) to show protein synthesis in healthy cells. Fluorescence data and time-constants could be analysed across each chamber to produce an 8 × 8 dataset for each individual chip, to compare different conditions. A toxic agent, cycloheximide, was applied across the device and the reduction in GFP activity could be quantitatively measured across an experimental duration of 60 h. Sub-lethal concentrations of cycloheximide would cause the GFP to decay, but could be recovered when perfused with fresh media. Different tubing was used across various tests to determine whether the leaching of gases through the plastic or pH changes would affect the results. When compared to tests that were run in static 96-well plates on tissue culture grade polystyrene and PDMS substrates, the datasets were found to be very similar in their decay time constants, but statistically superior in terms of their standard deviations and number of regions that could be analysed.

A three-dimensional flow cell microarray was produced by Eddings et al. [53], in which 48 individual flow cells were stacked in four rows of twelve, with individual inlets and outlets feeding each flow cell. Each individual microchannel was capable of being injected with a fluid, which could be spotted onto a sensor surface flush with the array head and electrochemically measured for the presence of certain analytes in solution, or used for the sequential patterning of ligands on the surface. The high-throughput nature of the three-dimensional flow cell array leads to the potential to use it in either “one on many” or “many on one” approaches with many different cell types, or with many different nanoparticles on a few cell types.

Wada et al. [54] demonstrated a cytotoxicity screening method using cells that were pre-transfected with a green fluorescent protein plasmid fused with a gene encoding for a heat-shock protein (HSP70B’), creating sensor cells that would express fluorescent protein biomarkers as the heat shock protein is expressed in the presence of cytotoxic compounds. They showed that these sensor cells could be bound and propagated inside a microfluidic channel to produce a near 100% coverage. Using laminar flow methods, the cells were exposed to an ionic cytotoxic compound (CdCl_2_) for 1 h in multiple concentrations with an in-line negative control area of 0% concentration, and could be assayed for a relative fluorescent signal compared to the negative control. They were able to produce a gradient-flow chip that used in-line mixing of CdCl_2_ and buffer solution to form eight unique concentrations in laminar streams that produced a fluorescence profile showing an increase in fluorescence with an increasing concentration of the cytotoxic compound. This technique shows that a pre-transfected cell line maybe very useful for high-throughput screening methods inside microchannels.

Cytotoxicity in anatomically relevant scenarios has been investigated by producing organ-on-a-chip devices where human cells are bound in a microfluidic channel or chamber. These are designed to mimic single or multiple human organs, and seek to provide information on specific diseases or cases of poor health. Gori et al. [55] produced a liver-on-a-chip device in which they were able to expose a 3D culture of healthy hepatic cells to solutions of free fatty acids, and observe the oxidative stress and resulting cytotoxicity from the overload of oils/fats. They proved that the liver cells could diffuse the nutrients and eliminate waste products due to being in the 3D culture environment, which leads to longer viability times and more accurate depictions of a true hepatic system compared to 2D analogous systems.

**Figure 3:**
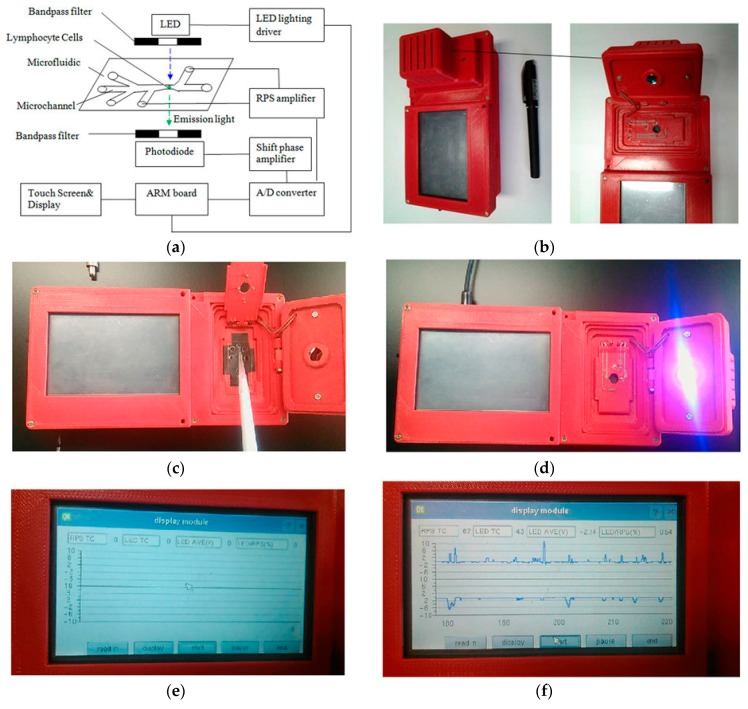
Handheld flow cytometer produced by Wang et al. (Scientific Reports, 6(2016) [56]), with miniaturized optical detectors, a disposable microfluidic analysis chip, and electronic display readouts. (**a**) schematic diagram; (**b**) device housing with space for microfluidic chip; (**c**) pipetting sample onto microfluidic chip; (**d**) UV LED active; (**e**) electronic display module; (**f**) electronic readout of sample electrophoretic data.

Some microfluidic devices do not rely on binding the cells to a specific area inside a microchannel, instead focusing on analyzing cells in suspension as they flow through the channel. The detection of biomarkers produced by ionizing radiation is often performed on expensive and complex flow cytometers, which reduces the amount of diagnoses available in remote or poorer areas. Also, in areas where radiation is particularly prevalent, such as power plants or space missions, tests should be performed to diagnose and prevent radiation sickness. In order to improve these issues, Wang et al. [56] produced a microfluidic device with a disposable chip that could perform the same fluorescence intensity readings for the most common radiation biomarker (γ-H2AX) in a hand-held format, shown in Figure 3, as well as provide information on the number of cells passing through the analysis area using a resistive pulse sensor (RPS) and thus obtain the ratio of damaged-to-undamaged cells. From this, they were able to analyze the extent of radiation damage from UV light on human lymphocytes, and obtained comparable results to a conventional flow cytometer. By designing the microfluidic channels with a detection spot or “sensing gate” of 15 μm and a channel height of only 30 μm, they were able to direct the cells to individually flow through the system under laminar flow for analysis. The device contained an on-board light emitting diode for exciting the fluorescent marker, and the software was able to acquire the data and produce readouts of the amount of radiation damage present in samples of lymphocyte cells sourced from anti-coagulant samples of human blood.

Recent advances in 3D printing technology have attempted to reduce the complexity of microfluidic fabrication and assembly. Devices with simple finger-tight joints have been created by groups such as Morgan et al. [57], who investigated not only the suitability of seeding encapsulated dental stem cells inside the printed microfluidics, but also optimized the transparency of the poly-lactic acid (PLA) polymer surface by controlling the printing parameters (layer thickness, print speed and fill patterning). This has been a major point of contention as to the usefulness of 3D printed structures for microfluidic devices, which require optically transparent flat surfaces for ideal imaging and analysis. Given that they were able to achieve this transparency, their testing was able to fluorescently visualize labelled cell aggregates and differentiate between live and dead cells. Thus, they are moving towards simplifying and standardizing the assembly of suitable microfluidics for toxicity testing, which will hopefully improve the speed at which they are considered, for more widespread use.

### 3.3. Droplet Microfluidics

**Figure 4:**
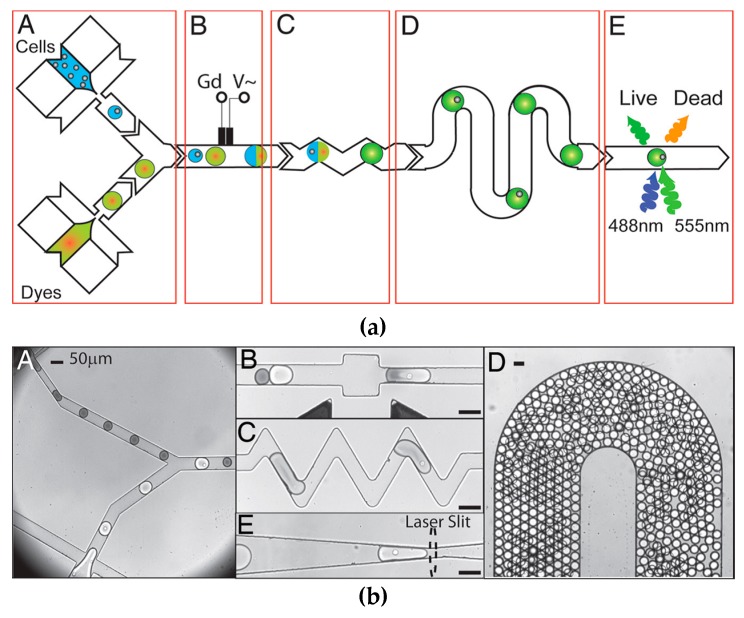
Schematic diagram and microscope images of viability assay by droplet generation reprinted with permission from Brouzes et al. Proc. Natl. Acad. Sci. USA., 106(34) (2009) Brouzes, Medkova [58], showing droplet fusion and reagent mixing, as well as high-throughput generation of cell-containing droplets. (**a**-**A**) Combination of cells and dyes, (**a**-**B**) in-line electrochemical sensor, (**a**-**C**) droplet fusion channels, (**a**-**D**) serpentine mixing channels (**a**-**E**) fluorescence assay. (**b**-**A**) micrograph of droplets entering channel, (**b**-**B**) droplet fusion, (**b**-**C**) droplet mixing, (**b**-**D**) high-throughput droplet generation in channel, (**b**-**E**) location of fluorescent excitation. Scale bars = 50 μm.

Microfluidic droplet generation is a technology whereby immiscible phases are combined to produce droplets that are stabilized by a carrier medium. The group of Brouzes et al. [58] demonstrated that single mammalian cells could be encapsulated in aqueous droplets, stabilized by an oil medium. When placed in an incubator, the encapsulated cells were found to stay viable for up to four days inside the droplet. They then flowed these cell-containing droplets in sequence with droplets of viability assay dyes, which were then mixed inside a well affected by an AC electrical field, causing electrically-controlled droplet fusion. This fused droplet was held for 15 min incubation on-chip, and could then be driven towards an in-line laser excitation and detection area that would determine the cell viability from the assay dye’s transmission wavelength. In this method, they were able to measure single-cell viabilities on a single human cell type after combining it with a library of optically labelled drug molecules. A histogram could then be produced, where each single-cell droplet showed a fluorescence signal corresponding to whether the encapsulated cell was alive or dead. These results show that this method could be used for the high-throughput screening of a large number of potential cytotoxic compounds, and could be scaled up to run many cell types in parallel. Figure 4 shows a schematic of their system, along with the microscope images of their on-chip procedure, and microscope image D shows that a large number of droplets can be produced in a single microfluidic environment, enabling a high-throughput of assay screening.

The group of Konry et al. [59] utilized droplet microfluidics to determine the cytotoxic effects of human immune system cells on cancer cells at the single cell level. By using the encapsulation method of flowing immiscible phases against each other, they were able to produce droplets with “distinct heterotypic cell pairs” and investigated the interactions of dendritic cells and T-lymphocytes. By introducing a cancer cell into the droplets, namely a multiple myeloma cell line, they were able to measure the speed at which CD8^+^ T-lymphocytes could achieve cytolysis of the foreign body and what effect different levels of antigen activation had on the time taken for cell death. They determined that the presence of interferon gamma, secreted by the myeloma cells, reduced the reactivity of the T-lymphocytes, and subsequently showed that the addition of a “neutralizing antibody” could prevent this loss of reactivity and improve the immune cells’ ability to kill off the cancer cells. As this technique is highly scalable, it could be used to test large variances in antigen concentration or different cell types in single runs.

## 4. Microfluidics for Nanotoxicity Screening

Microfluidic devices offer many advantages when it comes to cellular analysis with small sample volumes, reduced costs, controllability, and reproducibility. In addition, microfluidics offers the ability to introduce multiple biological conditions in a single device, and replicate in vivo conditions and dimensions. Thus, its usefulness in producing a high-throughput platform for toxicological experiments with nanomaterials is promising for screening applications. Small sample volumes can be very important when dealing with nanomaterial testing. Given that particulate matter may be produced from nanomaterials in extremely low concentrations, and that some nanomaterials are very expensive and produced in low quantities, there may be very limited amounts or diluted analytes to perform testing on. The behaviour of nanoparticles under flow is more difficult to quantify compared with that of macroscale particles. Therefore, the reproducible flow profiles and concentration gradients achieved in microfluidic nanotoxicity testing presents a distinct advantage.

Similar to cytotoxicity tests with other chemicals, the toxicity testing of nanomaterials is generally performed in bulk by seeding cells suspended in growth media into well plates using pipettes [17]. The cells are then exposed to nanomaterials in static conditions, which may cause the nanomaterials to adsorb or sediment onto the exposed surface of the cells under gravity [60]. Once these cells have been exposed to the nanomaterials for a certain time period, the cells can be assayed for their viability in a number of ways. Particular cell types may exhibit changed membrane permeability values and nanomaterial uptake properties when their morphology changes under flow, as compared to their sedentary morphologies, and thus, a static nanotoxicity test may provide inaccurate data.

**Figure 5:**
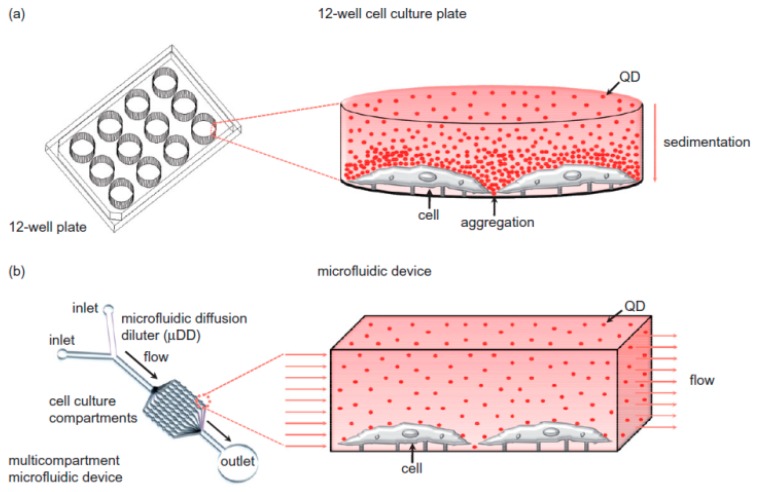
Diagram of nanoparticle behaviour inside well plates vs. microfluidic channel. (**a**) Well-plates without flow have particles aggregate under gravity, causing heterogeneous concentrations; (**b**) microfluidics can keep particles in homogenous suspension while under flow. Reprinted from Biomicrofluidics 4(2010) Mahto, Yoon [60], with the permission of AIP Publishing.

A review by Mahto et al. [61] goes into exceptional detail on the subject of nanomaterials in microfluidic environments, and brings up a number of important details regarding nanotoxicity. First, it refers to a number of studies that show that nanoparticles, depending on their size and shape, are passively taken up into nearly all cell types via endocytotic pathways [27,62,63]. Secondly, it refers to the potential pitfalls of current in vitro nanotoxicity testing methods. Notably, nanoparticles often react with the organic dyes commonly used in cell-based assays, meaning that they cannot be properly assayed [64]. Cell exposure to nanomaterials is often improperly controlled, with aggregation and sedimentation leading to very different exposure profiles (as seen in Figure 5). Current nanotoxicity testing also utilizes immortalized cell lines which differ significantly from primary human tissue [65]. The review article mentions platforms that look to circumvent the current issues with nanotoxicity testing, such as the device produced by Richter et al. [66], which used non-invasive electrodes to electrochemically measure the amount of collagen production as a label-free marker of cell viability. This device could detect the nanotoxicity of silver nanoparticles after 2 h of exposure, as compared to the lack of nanotoxicity seen for gold nanoparticles over a period of 24 h. It could also detect reductions in collagen production, given a sub-lethal concentration of silver nanoparticles. For further discussion, the authors recommend Mahto’s review paper to the reader.

The well-plate methodology for nanotoxicity screening has been debated for its suitability in replicating in vivo conditions [27,67,68,69,70]. This is investigated in detail in a paper by Mahto et al. [60], where nanoparticles influenced by gravity in a static system formed a concentration gradient within a cell culture plate. The static conditions involved in well-plate analysis, i.e., pipetting nanomaterial solutions on top of a cell culture and allowing them to sediment on top of the cellular layer, are thought to have limitations in providing accurate nanotoxicology data for cells that are under shear stress from flowing biological fluids such as arterial, lymphatic, and renal cells in vivo. To compare the differences between static and flowing nanoparticles, they tested a sample of core/shell CdSe/ZnSe quantum dots in static tissue culture plates for 12 h at 8–80 pM to discover the optimal cytotoxic range, and then exposed the same quantum dots through a microfluidic concentration gradient generator in cell culture medium to murine embryonic fibroblast cells. When exposed to the quantum dots under flow, the cells exhibited apoptosis effects, namely detachment and dose-dependent morphological changes. However, the difference between the two exposure conditions at 40 pM was significant, in that the static conditions showed higher percentages of cell death and increased cell deformities, suggested to be due to the physicochemical stress of the sedimentation of quantum dots onto the cell membranes.

The effect of the shear-stress effect of flowing media over cells has been investigated by groups such as Kim et al. [71], utilizing bound endothelial cells in a single microchannel and exposing them to mesoporous silica nanoparticles. The shear-stress forces were tuned to mimic those expected in the arterial and capillary system of a healthy human (5–6 N/m^2^), in order to observe any differences between these values and those of a static system. The nanoparticle concentration was also tuned to eliminate the effect of higher dosages during periods of a higher flow rate/higher shear-stress, so that the shear-stress forces were the primary variable. The unmodified silica nanoparticles used in this paper were found to increase in toxicity as the shear-stress increased, indicating an increase of cell membrane morphology and/or permeability under normal bodily shear-stress conditions, whereas the same dosages showed a reduced toxicity when applied in a static environment. This result suggests that static nanotoxicity tests may not be representative of the actual toxicity in a human body. If this is indeed the case, future toxicity tests would benefit from reproducing human vascular conditions in their flow and shear-stress properties.

In order to improve the cellular seeding and viability of hepatocytes in a microchannel, the group of Liu et al. [72] produced an electrospun biocompatible scaffold inside of a microfluidic device. By creating a 3D micro-environment of fibres for the liver cells, they were able to form a micro-perfusion environment which overcame the previous limitations of the lack of scaffold stiffness and the permeability to large molecules/cells. Using this platform, they were able to culture hepatocytes on the scaffold without microfluidic flow, then washed through and assayed for viability by measuring the albumin/urea secretion of the cells. The viability was determined to be higher under microfluidic perfusion than without perfusion flow or in static conditions. Upon the addition of silver nanoparticles, they could measure the amount of cell membrane damage with a commercial lactate dehydrogenase assay kit and found that the biomimetic 3D hepatocyte spheroids were more sensitive to silver nanoparticle damage than on a 2D tissue culture plate.

The use of cytometric methods can be integrated into microfluidic platforms to provide rapid and low cost nanotoxicity data, as was shown by Park et al. [73]. Their group cultured adherent cells (HeLa) directly into channels in a PDMS-glass microfluidic device and incubated the entire chip for 48 h to allow the cells to spread and grow over the analysis areas. They then introduced silver nanoparticles using a syringe pump via a concentration gradient generator. The silver nanoparticles induced both morphological changes in the cells and a colorimetric response to the MTT assay, which was also investigated in its conventional use in well-plates to compare against the microfluidic response. Optical brightfield images of the cell culture areas inside the channels were acquired post-exposure, to determine cell viability from the morphology and absorbance data. Dose-dependency was clearly observed for the toxicity of the silver nanoparticles and the half-lethal concentration (LC50) of the nanoparticles could be calculated. The LC50 from the microfluidic experiments was comparable to the estimated value from the conventional 96 well-plate method. The benefits of this technique using microfluidic approaches may include lower costs and the ease of use.

When single-cell responses to nanoparticle solutions are studied, using cell traps allows for very specific analyses to be performed on individual cells. Cunha-Matos et al. [67] formed cell traps designed to accept single cells inside a microfluidic channel using soft lithographic techniques, and then seeded the traps with functionalized gold nanorods, followed by Raman-active molecules and a coating of polyelectrolytes and proteins, that allowed them to bind primary bone marrow dendritic cells. The nanorods were then visible under surface-enhanced Raman spectroscopy (SERS), which allowed for the real-time visualization of nanoparticle concentration gradients as they were applied to the cells under flow. They used a live-cell incubator on a microscope stage to keep the cells in a biologically compatible environment for a duration of 24 h, over which they were able to assess each individual trapped cell for its response to nanoparticles. They were also able to add viability dyes to detect apoptosis and necrosis responses to the nanoparticles.

Adding electrodes to cell-trapping microwells via metallic deposition followed by chemical etching allows for electrophoretic measurements of cell viability. A thesis by Pratikkumar [74] detailed a microfluidic device that incorporates a combination of dielectrophoresis (DEP) and microwell methods of trapping single cells in wells aligned with gold microelectrodes, which allowed for the analysis of cells using electrochemical methods. The DEP forces could be switched on and off during the cell capture step, allowing the targeted capture of specific cells on the individual electrode/well features. Once the cells were captured, copper oxide nanoparticles were introduced into the microfluidic channel and flowed over the cell membranes to study the morphological response in each microwell. The author states that the impedance-based cell analysis was rapid, simple, label-free, and non-invasive. Measuring impedance vs. time revealed a significant drop in impedance after exposure to CuO nanoparticles. This correlated to a reduction in cell size and detachment from the electrode surface, which indicated a loss of viability due to toxic effects.

Similarly, a device containing impedance electrodes was also produced by the group of Rothbauer et al. [70], but with larger cell culture chambers instead of microwells. Human lung adenocarcinoma cells were cultured by on-chip perfusion in serum-containing media, until a confluent layer was formed across the electrode surface. Silica nanoparticles were administered under flow in serum-free media (in order to prevent contamination and a change in bioactivity of the nanoparticles). Serum-containing media was perfused again, in order to regenerate the tumour cells from their previous treatment. A metabolic assay was performed in parallel to the electrical impedance assay, and found that the AmSil30 silica nanoparticles caused a reduction in tumour regeneration and re-attachment to the electrode surface. Additionally, the presence of microfluidic flow in the device caused a reduction in regenerative capacity dependant on the flow velocity, indicating that the shear stress exerted on the cells played an important role in increasing the extent of nanoparticle uptake and thus the toxicity.

Organ-on-a-chip devices have only recently been used in nanotoxicity, and most often they utilize a single organ type. As their suitability for multi-organ toxicity assays becomes more fully realized, they will likely become more widely used. The group of Huh et al. [75] produced a lung-on-a-chip by seeding human alveolar epithelial cells and microvascular endothelial cells onto opposite sides of a porous PDMS membrane coated with an extracellular matrix protein. This membrane was sandwiched in between PDMS layers with large adjacent side channels, which were deformed by the application of a vacuum. This meant that the membrane could be subjected to mechanical stretching to simulate the action of breathing. The epithelial cells were exposed to air after their initial seeding was successful, while the endothelial cells remained exposed to culture medium with added blood-borne immune cells, providing an air-liquid interface to mimic the natural lung environment. In order to determine the device’s response to nanomaterials, a solution of 12 nm silica nanoparticles in fluid was injected over the epithelial layer and aspirated to leave a thin layer, mimicking an aerosol uptake of the solution. The silica nanoparticles were found to promote the inflammation of the underlying endothelial layer, seen by an increase in the expression of Intercellular Adhesion Molecule 1 (ICAM-1) and the increased capture of neutrophils, a type of white blood cell. This device was considered to have increased the efficacy due to the mechanical breathing motions, as the motion only promoted ICAM-1 production upon exposure to the silica nanoparticles. This finding suggests that a lung-on-a-chip device with a breathing motion may give more accurate results on nanotoxicity than a static culture.

## 5. Outlook

Microfluidics nanotoxicity screening offers a range of potential advantages over traditional screening methods. The ability to integrate parallel streams on the same chip allows for high-throughput screening in a small form factor, as well as the reduced use of reagents/analytes and a reduction in the overall testing time. However, microfluidic screening is not yet widely employed in nanotoxicity testing. This may be due to the many parameters that require optimization if an agreed standard operating procedure is to be broadly accepted.

Indeed, the standardization of testing is the most critical roadblock against the adoption of microfluidic nanotoxicity screening. In order to achieve this task, many fundamental studies on the interactions between nanomaterials and channel-bound cells must be performed. Parameters that must be defined include: the effects of channel dimension and flow rate on the amount of nanomaterial exposure; differences in exposure along the length of the channel; and potential run-on effects of affected cells upon downstream cells. The viability assay methods must also be normalized, whereas groups are currently researching multiple variations on dye assays, microscopy, and flow cytometry.

Currently, microfluidics for nanotoxicity screening faces a significant challenge, in that the adoption of a standard device design is perhaps the first necessary step to produce an accepted industry method. However, most research groups involved in the study of this field have their own unique ideas and designs for their devices. It is rare for a research group to precisely follow the designs of another in their own experiments, and therefore, most fundamental studies are performed on different platforms. It would require a concerted effort between multiple groups to agree on a design and perform the required standardization testing to make it suitable for commercialization, which has not yet occurred.

The fact that many different methodologies are still being researched indicates that each method may have its own merits. While droplet microfluidic methods do offer a substantial increase in the number of tests able to be performed in a single device, they tend to be more suited to single-cell or single-cluster analyses. The latter is of interest to fundamental studies of particle or drug uptake in cells that are often found alone or in small clusters. However, the segregation of cellular analysis is less favourable for toxicity screening. Adherent cells, for example, will not adopt the same layer configuration found in normal biology and would therefore give less meaningful data. To address this, researchers are turning to multi-organ labs-on-a-chip platforms. These 3D cellular environments are likely to achieve more biologically relevant discoveries, including any run-on effects of toxic species between cell types. Coupling these 3D cellular environments with microfluidic flow will facilitate the collection of uptake profiles of toxicants under conditions that mimic arterial shear stresses on cell types, i.e., better models of toxicity in the complex environment of the human body.

To summarize, it is likely that microfluidic technologies that are taken-up by the nanotoxicity screening industry will provide data that is extremely difficult to obtain through current well-plate methods. The most likely candidate for this is the organ-on-a-chip style of device, due to the close mimicry of the human body, as discussed above. If these platforms prove to be successful (biologically relevant), fast, accurate, and inexpensive, microfluidics-enabled nanotoxicity screening may become a widely accepted testing platform for industry and regulators alike.

## References

[1] Monica J.C., Heintz M.E., Lewis P.T. (2007). The perils of pre-emptive regulation. Nat. Nano.

[2] Bowman D.M., van Calster G., Friedrichs S. (2010). Nanomaterials and regulation of cosmetics. Nat. Nano.

[3] Balas F., Arruebo M., Urrutia J., Santamaria J. (2010). Reported nanosafety practices in research laboratories worldwide. Nat. Nano.

[4] Hunter R. (2014). In Vitro Toxicity Testing: Technologies and Global Markets.

[5] Alberts B., Johnson A., Lewis J., Raff M., Roberts K., Walter P. (2002). Molecular Biology of the Cell.

[6] Bianconi E., Piovesan A., Facchin F., Beraudi A., Casadei R., Frabetti F., Vitale L., Pelleri M.C., Tassani S., Piva F. (2013). An estimation of the number of cells in the human body. Ann. Hum. Biol..

[7] Vickaryous M.K., Hall B.K. (2006). Human cell type diversity, evolution, development, and classification with special reference to cells derived from the neural crest. Biol. Rev. Camb. Philos. Soc..

[8] Jani P., Halbert G.W., Langridge J., Florence A.T. (1990). Nanoparticle uptake by the rat gastrointestinal mucosa: Quantitation and particle size dependency. J. Pharm. Pharmacol..

[9] Penney D.P., Ferin J., Oberdorster G. (1992). Pulmonary retention of ultrafine and fine particles in rats. Am. J. Respir. Cell Mol. Biol..

[10] Wakefield G., Green M., Lipscomb S., Flutter B. (2004). Modified titania nanomaterials for sunscreen applications—Reducing free radical generation and DNA damage. Mater. Sci. Technol..

[11] Osmond M.J., McCall M.J. (2010). Zinc oxide nanoparticles in modern sunscreens: An analysis of potential exposure and hazard. Nanotoxicology.

[12] Mu L., Sprando R.L. (2010). Application of nanotechnology in cosmetics. Pharm. Res..

[13] Hu W., Peng C., Luo W., Lv M., Li X., Li D., Huang Q., Fan C. (2010). Graphene-Based Antibacterial Paper. ACS Nano.

[14] Li Q., Mahendra S., Lyon D.Y., Brunet L., Liga M.V., Li D., Alvarez P.J.J. (2008). Antimicrobial nanomaterials for water disinfection and microbial control: Potential applications and implications. Water Res..

[15] Marambio-Jones C., Hoek E.M.V. (2010). A review of the antibacterial effects of silver nanomaterials and potential implications for human health and the environment. J. Nanopart. Res..

[16] Betteridge D.J. (2000). What is oxidative stress?. Metabolism.

[17] Durán N., Guterres S.S., Alves O.L. (2013). Nanotoxicology: Materials, Methodologies, and Assessments.

[18] Finkel T., Holbrook N.J. (2000). Oxidants, oxidative stress and the biology of ageing. Nature.

[19] Chen Z., Meng H., Xing G., Chen C., Zhao Y., Jia G., Wang T., Yuan H., Ye C., Zhao F. (2006). Acute toxicological effects of copper nanoparticles in vivo. Toxicol. Lett..

[20] Meng H., Chen Z., Xing G., Yuan H., Chen C., Zhao F., Zhang C., Zhao Y. (2007). Ultrahigh reactivity provokes nanotoxicity: Explanation of oral toxicity of nano-copper particles. Toxicol. Lett..

[21] Ye Y., Liu J., Xu J., Sun L., Chen M., Lan M. (2010). Nano-SiO_2_ induces apoptosis via activation of p53 and Bax mediated by oxidative stress in human hepatic cell line. Toxicol. In Vitro.

[22] Ahamed M. (2013). Silica nanoparticles-induced cytotoxicity, oxidative stress and apoptosis in cultured A431 and A549 cells. Hum. Exp. Toxicol..

[23] Park E.-J., Yi J., Chung K.-H., Ryu D.-Y., Choi J., Park K. (2008). Oxidative stress and apoptosis induced by titanium dioxide nanoparticles in cultured BEAS-2B cells. Toxicol. Lett..

[24] Ko K.S., Kong I.C. (2014). Toxic effects of nanoparticles on bioluminescence activity, seed germination, and gene mutation. Appl. Microbiol. Biotechnol..

[25] Moran J.H., Schnellmann R.G. (1996). A rapid beta-NADH-linked fluorescence assay for lactate dehydrogenase in cellular death. J. Pharmacol. Toxicol. Methods.

[26] Zhao J., Castranova V. (2011). Toxicology of nanomaterials used in nanomedicine. J. Toxicol. Environ. Health Part B Crit. Rev..

[27] Wang T., Bai J., Jiang X., Nienhaus G.U. (2012). Cellular uptake of nanoparticles by membrane penetration: A study combining confocal microscopy with FTIR spectroelectrochemistry. ASC Nano.

[28] Kreyling W.G., Hirn S., Schleh C. (2010). Nanoparticles in the lung. Nat. Biotechnol..

[29] Guo Y., Terazzi E., Seemann R., Fleury J.B., Baulin V.A. (2016). Direct proof of spontaneous translocation of lipid-covered hydrophobic nanoparticles through a phospholipid bilayer. Sci. Adv..

[30] Kettiger H., Schipanski A., Wick P., Huwyler J. (2013). Engineered nanomaterial uptake and tissue distribution: From cell to organism. Int. J. Nanomed..

[31] Yang P.-H., Sun X., Chiu J.-F., Sun H., He Q.-Y. (2005). Transferrin-mediated gold nanoparticle cellular uptake. Bioconjug. Chem..

[32] Oh N., Park J.-H. (2014). Endocytosis and exocytosis of nanoparticles in mammalian cells. Int. J. Nanomed..

[33] Fischer H.C., Chan W.C.W. (2007). Nanotoxicity: The growing need for in vivo study. Curr. Opin.Biotechnol..

[34] Chiappini C., Almeida C. (2014). 8-Silicon nanoneedles for drug delivery. Semiconducting Silicon Nanowires for Biomedical Applications.

[35] Kolhar P., Doshi N., Mitragotri S. (2011). Polymer nanoneedle-mediated intracellular drug delivery. Small.

[36] Carmona H., Valadez H., Yun Y., Sankar J., Estala L., Gomez F.A. (2015). Development of microfluidic-based assays to estimate the binding between osteocalcin (BGLAP) and fluorescent antibodies. Talanta.

[37] Hosokawa M., Hayashi T., Mori T., Yoshino T., Nakasono S., Matsunaga T. (2011). Microfluidic device with chemical gradient for single-cell cytotoxicity assays. Anal. Chem..

[38] Ng E., Hoshino K., Zhang X. (2013). Microfluidic immunodetection of cancer cells via site-specific microcontact printing of antibodies on nanoporous surface. Methods.

[39] Pasirayi G., Scott S.M., Islam M., O’Hare L., Bateson S., Ali Z. (2014). Low cost microfluidic cell culture array using normally closed valves for cytotoxicity assay. Talanta.

[40] Cunha-Matos C.A., Millington O.R., Wark A.W., Zagnoni M. (2016). Real-time assessment of nanoparticle-mediated antigen delivery and cell response. Lab Chip.

[41] Weibull E., Matsui S., Sakai M., Andersson Svahn H., Ohashi T. (2013). Microfluidic device for generating a stepwise concentration gradient on a microwell slide for cell analysis. Biomicrofluidics.

[42] Tong Z., Ivask A., Guo K., McCormick S., Lombi E., Priest C., Voelcker N.H. (2017). Crossed flow microfluidics for high throughput screening of bioactive chemical-cell interactions. Lab Chip.

[43] Roth E.A., Xu T., Das M., Gregory C., Hickman J.J., Boland T. (2004). Inkjet printing for high-throughput cell patterning. Biomaterials.

[44] Melamed S., Elad T., Belkin S. (2012). Microbial sensor cell arrays. Curr. Opin. Biotechnol..

[45] Leclerc E., El Kirat K., Griscom L. (2008). In situ micropatterning technique by cell crushing for co-cultures inside microfluidic biochips. Biomed. Microdevices.

[46] Wu J., Chen Q., Liu W., He Z., Lin J.-M. (2017). Recent advances in microfluidic 3D cellular scaffolds for drug assays. TrAC Trends Anal. Chem..

[47] Toh Y.-C., Lim T.C., Tai D., Xiao G., van Noort D., Yu H. (2009). A microfluidic 3D hepatocyte chip for drug toxicity testing. Lab Chip.

[48] Yoo S.K., Lee J.H., Yun S.-S., Gu M.B., Lee J.H. (2007). Fabrication of a bio-MEMS based cell-chip for toxicity monitoring. Biosens. Bioelectron..

[49] Wagner I., Materne E.M., Brincker S., Süßbier U., Frädrich C., Busek M., Sonntag F., Sakharov D.A., Trushkin E.V., Tonevitsky A.G. (2013). A dynamic multi-organ-chip for long-term cultivation and substance testing proven by 3D human liver and skin tissue co-culture. Lab Chip.

[50] SynVivo SynTox 3D Toxicology Model—Organ Specific Physiological Responses: SynVivo. http://www.synvivobio.com/syntox/.

[51] Ma B., Zhang G., Qin J., Lin B. (2009). Characterization of drug metabolites and cytotoxicity assay simultaneously using an integrated microfluidic device. Lab Chip.

[52] Cooksey G.A., Elliott J.T., Plant A.L. (2011). Reproducibility and Robustness of a Real-Time Microfluidic Cell Toxicity Assay. Anal. Chem..

[53] Eddings M.A., Eckman J.W., Arana C.A., Papalia G.A., Connolly J.E., Gale B.K., Myszka D.G. (2009). “Spot and hop”: Internal referencing for surface plasmon resonance imaging using a three-dimensional microfluidic flow cell array. Anal. Biochem..

[54] Wada K.-I., Taniguchi A., Kobayashi J., Yamato M., Okano T. (2007). Live Cells-Based Cytotoxic Sensorchip Fabricated in a Microfluidic System. Biotechnol. Bioeng..

[55] Gori M., Simonelli M.C., Giannitelli S.M., Businaro L., Trombetta M., Rainer A. (2016). Investigating Nonalcoholic Fatty Liver Disease in a Liver-on-a-Chip Microfluidic Device. PLoS ONE.

[56] Wang J., Fan Z., Zhao Y., Song Y., Chu H., Song W., Song Y., Pan X., Sun Y., Li D. (2016). A new hand-held microfluidic cytometer for evaluating irradiation damage by analysis of the damaged cells distribution. Sci. Rep..

[57] Morgan A.J.L., Hidalgo San Jose L., Jamieson W.D., Wymant J.M., Song B., Stephens P., Barrow D.A., Castell O.K. (2016). Simple and versatile 3D printed microfluidics using fused filament fabrication. PLoS ONE.

[58] Brouzes E., Medkova M., Savenelli N., Marran D., Twardowski M., Hutchinson J.B., Rothberg J.M., Link D.R., Perrimon N., Samuels M.L. (2009). Droplet microfluidic technology for single-cell high-throughput screening. Proc. Natl. Acad. Sci..

[59] Konry T., Sarkar S., Sabhachandani P., Stroopinsky D., Palmer K., Cohen N., Rosenblatt J., Avigan D., Konry T. (2016). Dynamic analysis of immune and cancer cell interactions at single cell level in microfluidic droplets. Biomicrofluidics.

[60] Mahto S.K., Yoon T.H., Rhee S.W. (2010). A new perspective on in vitro assessment method for evaluating quantum dot toxicity by using microfluidics technology. Biomicrofluidics.

[61] Mahto S.K., Charwat V., Ertl P., Rothen-Rutishauser B., Rhee S.W., Sznitman J. (2015). Microfluidic platforms for advanced risk assessments of nanomaterials. Nanotoxicology.

[62] Geiser M., Rothen-Rutishauser B., Kapp N., Schürch S., Kreyling W., Schulz H., Semmler M., Hof V.I., Heyder J., Gehr P. (2005). Ultrafine particles cross cellular membranes by nonphagocytic mechanisms in lungs and in cultured cells. Environ. Health Perspect..

[63] Rothen-Rutishauser B.M., Schurch S., Haenni B., Kapp N., Gehr P. (2006). Interaction of fine particles and nanoparticles with red blood cells visualized with advanced microscopic techniques. Environ. Sci. Technol..

[64] Balbus J.M., Maynard A.D., Colvin V.L., Castranova V., Daston G.P., Denison R.A., Dreher K.L., Goering P.L., Goldberg A.M., Kulinowski K.M. (2007). Meeting report: Hazard assessment for nanoparticles--report from an interdisciplinary workshop. Environ. Health Perspect..

[65] Nel A.E., Madler L., Velegol D., Xia T., Hoek E.M.V., Somasundaran P., Klaessig F., Castranova V., Thompson M. (2009). Understanding biophysicochemical interactions at the nano-bio interface. Nat. Mater..

[66] Richter L., Charwat V., Jungreuthmayer C., Bellutti F., Brueckl H., Ertl P. (2011). Monitoring cellular stress responses to nanoparticles using a lab-on-a-chip. Lab Chip.

[67] Cunha-Matos C.A., Millington O.M., Wark A.W., Zagnoni M. Real-Time Multimodal Imaging of Nanoparticle-Cell Interactions in High-Throughput Microfluidics. Proceedings of the 18th International Conference on Miniaturized Systems for Chemistry and Life Sciences.

[68] Velve-Casquillas G., Le Berre M., Piel M., Tran P.T. (2010). Microfluidic tools for cell biological research. Nano Today.

[69] Shah P., Kaushik A., Zhu X., Zhang C., Li C.-Z. (2014). Chip based single cell analysis for nanotoxicity assessment. Analyst.

[70] Rothbauer M., Praisler I., Docter D., Stauber R.H., Ertl P. (2015). Microfluidic impedimetric cell regeneration assay to monitor the enhanced cytotoxic effect of nanomaterial perfusion. Biosensors.

[71] Kim D., Lin Y.-S., Haynes C.L. (2012). On-chip evaluation of shear stress effect on cytotoxicity of mesoporous silica nanoparticles. Anal. Chem..

[72] Liu Y., Wang S., Wang Y. (2016). Patterned fibers embedded microfluidic chips based on PLA and PDMS for Ag nanoparticle safety testing. Polymers.

[73] Park J.Y., Yoon T.H. (2012). Microfluidic image cytometry (μFIC) assessments of silver nanoparticle cytotoxicity. Bull. Korean Chem. Soc..

[74] Shah P. (2014). Development of a Lab-on-a-Chip Device for Rapid Nanotoxicity Assessment In Vitro. Ph.D. Thesis.

[75] Huh D., Matthews B.D., Mammoto A., Montoya-Zavala M., Hsin H.Y., Ingber D.E. (2010). Reconstituting organ-level lung functions on a chip. Science.

